# Gut microbial indicators of metabolic health underlie age-related differences in obesity and diabetes risk among Native Hawaiians and Pacific Islanders

**DOI:** 10.3389/fcimb.2022.1035641

**Published:** 2022-12-21

**Authors:** Riley K. Wells, Braden P. Kunihiro, Krit Phankitnirundorn, Rafael Peres, Trevor A. McCracken, Lesley Umeda, Rosa H. Lee, Dong Yoon Kim, Ruben Juarez, Alika K. Maunakea

**Affiliations:** ^1^ Department of Molecular Biosciences and Bioengineering, University of Hawaii at Manoa, Honolulu, HI, United States; ^2^ Department of Anatomy, Biochemistry, and Physiology, John A. Burns School of Medicine, Honolulu, HI, United States; ^3^ Institutional Development Awards (IDeA) Networks of Biomedical Research Excellence (INBRE), University of Hawaii at Manoa, Honolulu, HI, United States; ^4^ Department of Economics, University of Hawaii at Manoa, Honolulu, HI, United States; ^5^ University of Hawaii Economic Research Organization (UHERO), University of Hawaii at Manoa, Honolulu, HI, United States

**Keywords:** Native Hawaiian, age, microbiome, diabetes, body mass index, obesity

## Abstract

Native Hawaiians and Pacific Islanders (NHPIs) suffer from higher prevalence of and mortality to type 2 diabetes mellitus (T2DM) than any other major race/ethnic group in Hawaii. Health inequities in this indigenous population was further exacerbated by the SARS-CoV-2 pandemic. T2DM progression and medical complications exacerbated by COVID-19 are partially regulated by the gut microbiome. However, there is limited understanding of the role of gut bacteria in the context of inflammation-related diseases of health disparities including T2DM and obesity. To address these gaps, we used a community-based research approach from a cohort enriched with NHPI residents on the island of Oahu, Hawaii (N=138). Gut microbiome profiling was achieved *via* 16s rDNA metagenomic sequencing analysis from stool DNA. Gut bacterial capacity for butyrate-kinase (BUK)-mediated fiber metabolism was assessed using quantitative PCR to measure the abundance of *BUK* DNA and RNA relative to total bacterial load per stool sample. In our cohort, age positively correlated with hemoglobin A1c (%; *R=0.39; P<0.001*) and body mass index (BMI*; R=0.28; P<0.001*). The relative abundance of major gut bacterial phyla significantly varied across age groups, including *Bacteroidetes (P<0.001), Actinobacteria (P=0.007)*, and *Proteobacteria (P=0.008)*. A1c was negatively correlated with the relative levels of *BUK* DNA copy number *(R=-0.17; P=0.071)* and gene expression *(R=-0.33; P=0.003)*. Interestingly, we identified specific genera of gut bacteria potentially mediating the effects of diet on metabolic health in this cohort. Additionally, α-diversity among gut bacterial genera significantly varied across T2DM and BMI categories. Together, these results provide insight into age-related differences in gut bacteria that may influence T2DM and obesity in NHPIs. Furthermore, we observed overlapping patterns between gut bacteria and T2DM risk factors, indicating more nuanced, interdependent interactions among these factors as partial determinants of health outcomes. This study adds to the paucity of NHPI-specific data to further elucidate the biological characteristics associated with pre-existing health inequities in this racial/ethnic group that is significantly underrepresented in biomedical research.

## 1 Introduction

Native Hawaiians and Pacific Islanders (NHPIs) suffer from a disproportionately higher prevalence of and mortality to type 2 diabetes mellitus (T2DM) than any other major race/ethnic group in Hawaii (Hawaii State Department of Health, 2019). In 2018, the age-adjusted diabetes death rate among NHPIs was reported at 48.1% - over 2.5 times higher than that of the general state population (Centers for Disease Control and Prevention [CDC], 2018). The following year, NHPIs accounted for 31.2% of reported diabetic cases among Hawaii residents. T2DM prevalence among NHPIs increases annually at a rate comparably faster than that of other racial/ethnic groups in the state (Uchima et al., 2019). In addition, this pre-existing health disparity has been implicated as a determinant of the heightened risk to severe COVID-19 (Gregory et al., 2020; Penaia et al., 2021; Zhou et al., 2021).

Given that health disparities including T2DM and severe COVID-19 are also compounded by data disparities arising from underrepresentation of NHPIs and other minority race/ethnic populations in biomedical research (McElfish et al., 2019; Uchima et al., 2019; Wang et al., 2020; Kamaka et al., 2021: Penaia et al., 2021), understanding the relationships between established or emerging biomarkers and anthropometric data relevant to cardiometabolic health in these populations are urgently needed. There are several established and emerging clinical indicators of cardiometabolic health, however the degree of variability of these indicators and their relationship to T2DM risk remain understudied in the NHPI population (Kamaka et al., 2021). Of the established indicators, the percentage of glycosylated hemoglobin A1c in blood has now become a wide-spread, point-of-care diagnostic standard for T2DM, where A1c levels are stratified into non-diabetic (less than 5.7%), pre-diabetic (between 5.7% and 6.5%), and diabetic (greater than 6.5%) categories (American Diabetes Association, 2021). Body mass index (BMI; kg/m^2^) scores are similarly considered indicative of cardiometabolic health upon stratification into normal (less than 25), overweight (between 25 and 30), and obese (greater than 30) categories (CDC, 2021). However, the applicability of BMI as an indicator of cardiometabolic health outcomes has been demonstrated to vary with age and ethnicity (Uchima et al., 2019).

A developing indicator of cardiometabolic health of increasing interest to clinical and biomedical research studies is the gut microbiome (Everard and Cani, 2013; Panwar et al., 2013; Tilg and Moschen, 2014; Upadhyaya and Banerjee, 2015; Gomez-Arango et al., 2016; Wen and Duffy, 2017; Chen et al., 2021). Recent metagenome-wide association studies have implicated the bidirectional relationship between the gut microbiome and host physiology as a partial determinant of cardiometabolic health outcomes (Tilg and Moschen, 2014; Upadhyaya and Banerjee, 2015; Chen et al., 2021). Exploratory, diet-based interventions leveraging this relationship have demonstrated sufficiency in enhancing blood glucose homeostasis (Panwar et al., 2013). This phenomenon is partially mediated by serum metabolites originating from gut microbes, many of which have been identified as direct determinants of T2DM pathophysiology and adjacent complications (Arpaia et al., 2013; Panwar et al., 2013; Wen and Duffy, 2017; Wang et al., 2018; Chen et al., 2021).

One such metabolite is butyrate, a short-chain fatty acid (SCFA) byproduct of bacterial fiber metabolism (Roediger, 1980; Arpaia et al., 2013; Menni et al., 2020). While prior studies have reported a negative relationship between the relative abundance of butyrate-producing bacteria and T2DM risk (Arora and Tremaroli, 2021), dynamic interactions among gut bacterial butyrate metabolism and cardiometabolic health outcomes in NHPIs is unclear (De la Cuesta-Zuluaga et al., 2019). Dietary adjustment is a non-invasive therapeutic strategy for modulating gut microbiome dynamics (Panwar et al., 2013; Karmally and Goldberg, 2021). Fiber-rich dietary tendencies have been demonstrated to reduce A1c levels (Panwar et al., 2013; Fu et al., 2022) and systemic inflammation (Nastasi et al., 2015; Wen and Duffy, 2017; Scheithauer et al., 2020), suggested in reducing T2DM risk. However, the relationship among dietary habits, gut bacterial population dynamics, and T2DM risk factors in NHPI populations is unknown and hinders efforts to comprehensively understand T2DM and obesity health disparities in this population (Kamaka et al., 2021). To address this gap in knowledge, we characterized the variability of the gut microbiome in a NHPI-enriched cohort in Hawaii with extensive anthropometric and other health-related data.

## 2 Materials and methods

### 2.1 Human subjects data collection

IRB approval was obtained from the University of Hawaii Institutional Review Board (UH IRB) under protocol number 2019-00376. All laboratory procedures were evaluated and approved by the Hawaii Institutional Biosafety Committee, under protocol number B22-100652.

Participants included in this cohort study (N=138; aged 16 to 79 years) mainly resided in one of two NHPI-enriched communities on Oahu, Hawaii (Waianae and Palolo). Informed consent, sociodemographic information, medical history, and behavioral risk factor data were collected using self-reported survey responses. Personal information was de-identified for each participant through assignment of unique numerical ID. Biometric data including height, weight, and A1c (PTS Diagnostics, Whitestown, IN) were collected upon survey completion. T2DM categories (non-diabetic, pre-diabetic, diabetic) and BMI categories (healthy, overweight, obese) were defined using A1c and BMI value cut points recommended by the American Diabetes Association (ADA) and the Centers for Disease Control and Prevention (CDC), respectively.

### 2.2 Stool sample storage and processing

Home stool sample self-collection kits were distributed to participants upon biometric data collection. Each kit included one sample tube containing RNAlater (5 ml; a sample preservative supplied by Thermofisher Scientific, Waltham, MA). Instructions for proper sample collection and storage (-20°C) were provided verbally and in print. Samples were submitted by mail or collected by a community facilitator within one week of biometric data collection.

**Figure 1:**
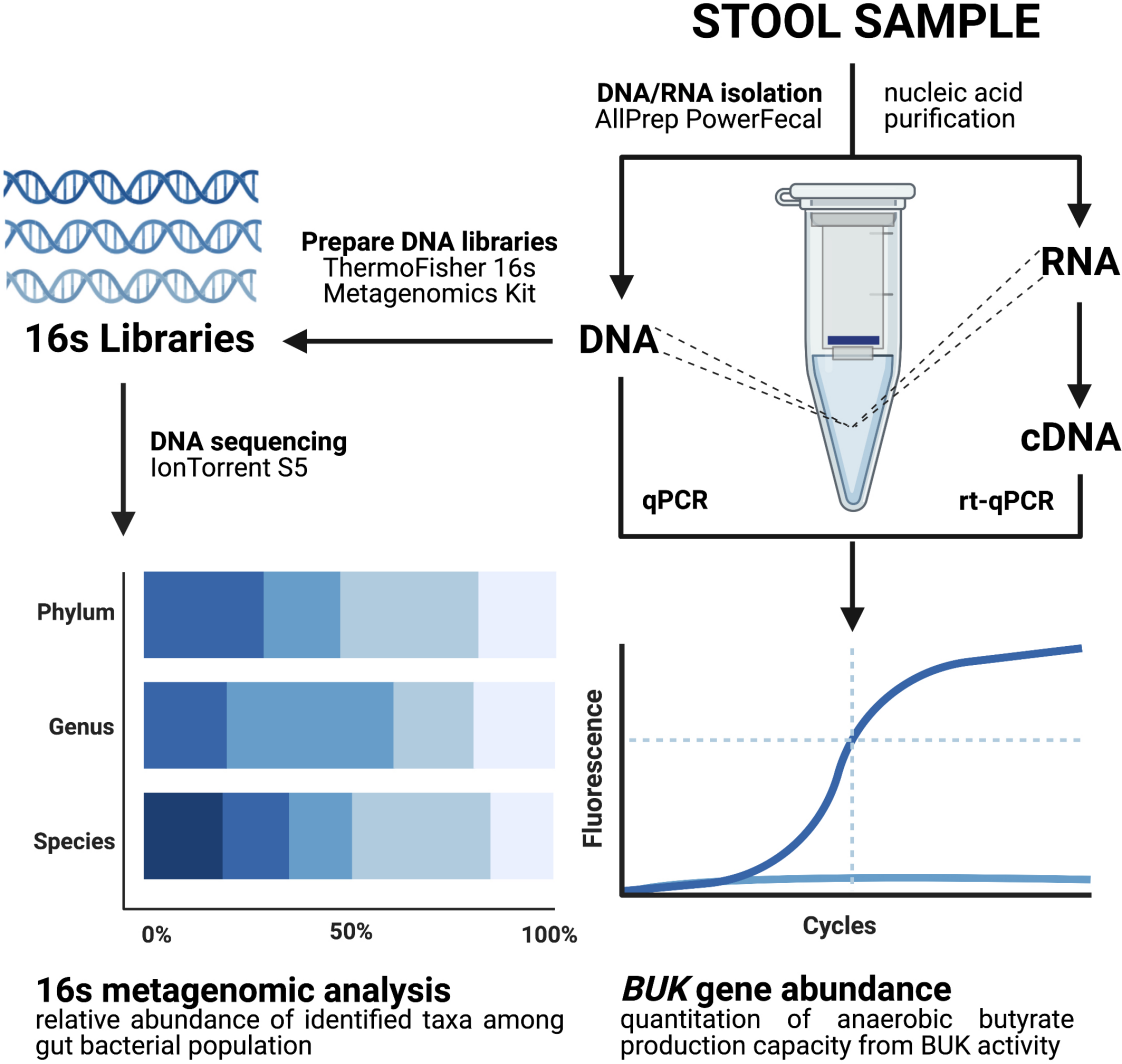
Overview of methodology for stool sample processing.

DNA and RNA were simultaneously isolated using the AllPrep PowerFecal DNA/RNA Kit (Qiagen Inc., Valencia, CA, USA) and stored in -80°C until further processing. Quality and concentration of nucleic acid yields were assessed using the NanoDrop Microvolume Spectrophotometer (ThermoFisher Scientific, Waltham, MA, USA). An overview of workflow methodology for stool sample processing and analyses are illustrated in **Figure 1**.

### 2.3 16s metagenomic sequencing

**Figure 2:**
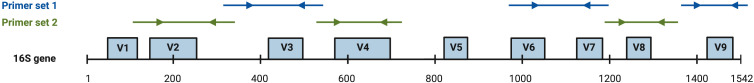
ThermoFisher 16s rDNA hypervariable region (hvr) proprietary PCR primer coverage (Barb et al., 2016). Arrows are representative of target binding sites and directionality of amplification. Primer set 1 achieves amplification of hvr V3, V6, V7, and V9. Primer set 2 achieves amplification of hvr V2, V4, and V8.

DNA (40 ng) isolated from each stool sample were subjected to polymerase chain reaction (PCR) amplification targeting 16s rDNA hypervariable regions V2-4 and V6-9 (**Figure 2**; Ion Torrent 16s Metagenomics Kit; Thermo Fisher Scientific, Warrington, England).

Amplicon products were pooled (20 uL per primer set), purified (Agencourt Ampure XP Kit; Beckman Coulter, Brea, CA, USA), and quantified using the Qubit dsDNA BR Assay (ThermoFisher Scientific, Warrington, England). 16s rDNA libraries were prepared from 150 ng of pooled amplicons (Ion Plus Fragment Library Kit; Thermo Fisher Scientific, Austin, TX, USA) and barcoded using Ion Xpress Barcode Adapters (Life Technologies, Carlsbad, CA, USA). DNA libraries were pooled (80 pmol from up to 60 libraries) and loaded onto Ion 530™ chips (Ion S5 Next-Generation Sequencing System) in preparation for sequencing.

16s Metagenomics Kit analysis was performed using Ion Reporter™ Software v5.18.4.0 (ThermoFisher Scientific). Chimeric sequences were automatically identified and removed. Reads were mapped to reference databases Greengenes v13.5 and MicroSEQ ID v3.0. Gut microbiome profiles were compiled using metagenome taxonomic data *via* the Curated MicroSEQ(R) 16S Reference Library v2013.1. Raw abundance values were subsampled at 10,000 reads per sample to control for inequivalent read numbers across samples. Subsampling was performed on the species-level operational taxonomic unit (OTU) table *via* the *rrarefy* function of the *vegan* R package (Oksanen et al., 2022). Samples with less than 10,000 total reads were excluded from the dataset. Upstream taxonomic ranks were determined by systematically comparing family-level OTU data to the NCBI database *via* the classification function of the *taxize* R package (Chamberlain et al., 2020). Family-level OTU table was the preferred classification input due to large amounts of unclassified upstream classifications when using genus and species-level OTU tables. Genus and species-level OTU tables were joined onto the family-OTU table to form a comprehensive taxonomic classification. Subsampled reads on the species-level were converted to per-sample relative abundance values *via* the “transform” function of the microbiome R package (Lahti and Shetty, 2019). Shannon, Simpson and Chao-1 α-diversity values were computed *via* IonReporter v5.18.4.0.

### 2.4 Measuring *BUK-*mediated butyrate metabolism

RNA (40 ng per sample) isolated from stool specimens were converted to cDNA using SuperScript IV VILO Master Mix with ezDNase™ Enzyme (Thermo Fisher Scientific, Waltham, MA, USA). PCR primers were synthesized (Thermo Fisher Scientific, Custom DNA Oligos Synthesis Services, Waltham, MA, USA) using butyrate kinase (*BUK*) as a gene target. DNA and cDNA yields (20 uL per sample) were subjected to quantitative PCR (qPCR; PerfeCTa SYBR Green FastMix, Quantabio, Beverly, MA, USA) in duplicate reactions on 96-well plates using the StepOnePlus Real-Time PCR instrument (Thermo Fisher Scientific, Waltham, MA, USA). Thermal cycling was programmed as follows: 95°C for 2 min; 40 cycles of 55°C for 15 sec followed by 72°C for 1 min with a hold at 4°C for later storage. Target concentration was calculated using qPCR using the delta-Ct method.

Gut bacterial SCFA metabolic pathways primarily result in synthesis of acetate, propionate, and butyrate (Louis and Flint, 2017). Three bacterial enzymes have been identified as main catalysts for the final step in butyrate synthetic pathways: acetyl-Coenzyme A (CoA):acetoacetyl-CoA transferase (ATO), butyryl-CoA:acetate CoA-transferase (BUT), and butyrate kinase (BUK) (Anand et al., 2016). While expression and activity of any of these three enzymes may provide insight into bacterial butyrate production capacity, CoA-transferases commonly exhibit broad substrate specificity (Louis and Flint, 2017). BUT isolated from *Roseburia hominis* serves as a relevant example of this phenomenon, where although it has been identified as the bacterium’s primary catalyst for butyrate production, its dual affinity for propionyl-CoA and butyryl-CoA enable participation in multiple SCFA synthetic pathways (Charrier et al., 2006).

Although microenvironmental conditions may shift enzymatic substrate preferences for butyrate-producing CoA-transferases BUT and ATO (Diez-Gonzalez et al., 1999; Sauer and Eikmanns, 2005; Sato et al., 2016), BUK participates in butyrate synthesis *via* the hydrolytic conversion of a phosphorylated intermediate butyryl-phosphate (Eeckhaut et al., 2011; Anand et al., 2016). While a similar reaction step is mediated by acetate kinase (ACK; Hartmanis and Gatenbeck, 1984), BUK is distinct from ACK in its substrate specificity for butyryl-phosphate rather than other SCFA precursors or long-chain fatty acid (LCFA) precursors (Sirobhushanam et al., 2017; Bui et al., 2021; Imdad et al., 2022). For the purpose of this study, we focused on measuring the abundance of *BUK* gene copies and transcriptional products to investigate butyrate metabolism and its relationship with T2DM-related health outcomes among NHPI communities.

*BUK* qPCR primers were designed as previously described by Gomez-Arango et al., 2016: Forward: 5′ - TGCTGTWGTTGGWAGAGGYGGA - 3′; Reverse: 5′ - GCAACIGCYTTTTGATTTAATGCATGG - 3’.

*BUK* quantitation was normalized against concurrent amplification by universal 16s qPCR primers (labeled *“BAC”*) as a proxy for total bacterial load. *BAC* primers were designed as previously described by Castillo et al., 2006: 63F: 5′ - GCAGGCCTAACACATGCAAGTC - 3′; 355R: 5′ - CTGCTGCCTCCCGTAGGAGT - 3’

### 2.5 Data analysis

Intergroup comparisons of nominal distributions of biometric data were performed using Kruskal-Wallis one-way analysis of variance (ANOVA), followed by Dunn’s Multiple Comparison Test. P-values for multiple comparisons were adjusted *via* the Benjamini-Hochberg method. Categorical distributions were analyzed using Pearson’s chi-squared tests for independence. Relationships between variables were measured using Spearman rank-order correlation coefficients (R Core Team, 2021). Data was visualized *via ggplot2* and *ggpubr* packages (Kassambara, 2020). Due to conceptual and technical limitations around integration of molecular and anthropometric variables, and sample size, statistical significance was determined at *P<0.05* for molecular data analyses, and at *P<0.10* for exploratory analyses with anthropometric variables. A flowchart describing quality control and inclusion/exclusion criteria used to arrive at the reported dataset is provided in **Figure S1**.

## 3 Results

### 3.1 Dynamic interactions among T2DM risk factors in NHPIs

**Figure 3:**
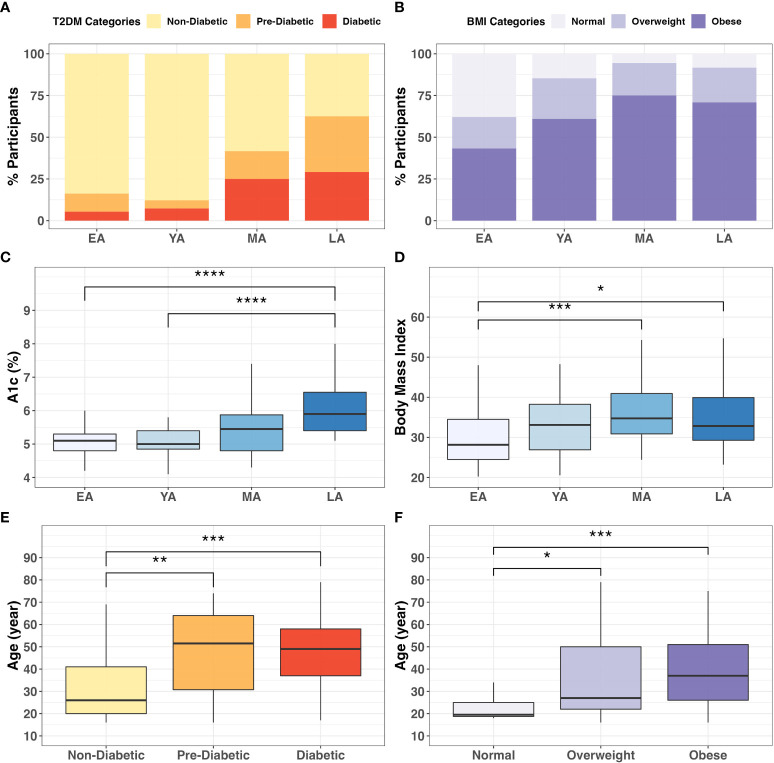
Categorical associations between age, T2DM, and BMI. The proportion individuals in the cohort in **(A)** T2DM (*X^2 =^ 27.6; P<0.001*) and **(B)** BMI categories (X^2 =^ *17.8; P<0.001)* varied significantly across age groups. Intergroup comparison demonstrated significantly different **(C)** A1c *(P<0.0001)* and **(D)** BMI *(P=0.005)* values across age groups. Additionally, participant age significantly differed across **(E)** T2DM *(P<0.001)* and **(F)** BMI *(P=0.001)* categories. * = P<0.05; ** = P<0.01; *** = P<0.001; ****= P<0.0001.

Descriptive statistics summarizing our NHPI-enriched cohort are provided in **Table 1**. To control for considerable variance in age among participants (*σ^2^= 288.3*), the total study population was subdivided into four age groups: Early Adulthood (EA; 16-20 years), Young Adulthood (YA; 21-35 years), Mid-Adulthood (MA; 36-55 years), and Late Adulthood (LA; 56+ years). Individual membership to T2DM (**Figure 3A** **;** *X^2^* =*25.5, P<0.001)* and BMI (**Figure 3B** **;** *X^2^=16.9, P=0.010*) categories significantly varied across age groups. Furthermore, differences between EA-YA and MA-LA individuals were observed for both A1c (**Figure 3C**) and BMI (**Figure 3D**). A1c (*P<0.001)* and BMI *(P=0.005)* values both differed between age groups, increasing significantly across successively older groups.

**Table 1:** Sociodemographic and biometric summary of the NHPI-enriched cohort stratified by age into four groups: Early Adulthood (EA; aged 16-20 years), Young Adulthood (YA; aged 21-35 years), Mid-Adulthood (MA; aged 36-55 years), and Late Adulthood (LA; aged 56-80 years).

	Cohort	Age Groups				
		EA	YA	MA	LA	P^a^
**Participants** (N; %)	138	37 (26.8%)	41 (29.7%)	36 (26.1%)	24 (17.4%)	
**Sex** (N; %)					X^2^ = 7.5	0.058^b^
Male	61 (44.2%)	23 (62.2%)	14 (34.1%)	13 (36.1%)	11 (45.8%)	
Female	77 (55.8%)	14 (37.8%)	27 (65.9%)	23 (63.9%)	13 (54.2%)	
**BMI** (mean ± SE)	33.3 ± 0.77	29.0 ± 1.23	34.6 ± 1.65	35.1 ± 1.29	35.3 ± 1.81	**0.005** ^a^
**BMI categories** (N; %)					X^2^ = 16.9	**0.010** ^b^
Normal	24 (17.4%)	14 (37.8%)	6 (14.6%)	2 (5.6%)	2 (8.3%)	
Overweight	29 (21.0%)	7 (18.9%)	10 (24.4%)	7 (19.4%)	5 (20.8%)	
Obese	85 (61.6%)	16 (43.2%)	25 (61.0%)	27 (75.0%)	17 (70.8%)	
**A1c** (mean ± SE)	5.7 ± 0.13	5.4 ± 0.21	5.3 ± 0.19	6.1 ± 0.31	6.4 ± 0.27	**<0.001** ^a^
**T2DM categories** (N; %)					X^2^ = 25.5	**<0.001** ^b^
Non-diabetic	97 (70.3%)	31 (83.8%)	36 (87.8%)	21 (58.3%)	9 (37.5%)	
Pre-diabetic	20 (14.5%)	4 (10.8%)	2 (4.9%)	6 (16.7%)	8 (33.3%)	
Diabetic	21 (15.2%)	2 (5.4%)	3 (7.3%)	9 (25.0%)	7 (29.2%)	

Bold P-values indicate statistical significance at α=0.05.

a Kruskal-Wallis ANOVA.

b Pearson’s chi-squared test for independence.

These differences were verified by direct intergroup comparisons for T2DM (**Figure 3E**; *P<0.001*) and BMI (**Figure 3F** **;** *P=0.001*) categories. In our cohort, age increased across successive T2DM categories, as diabetics (*P<0.001)* and pre-diabetics (*P<0.01)* tended to be older than non-diabetic individuals (**Figure 3E**). Similarly, individuals in the obese *(P<0.001)* and overweight (*P<0.01)* BMI categories tended to be older than individuals with normal BMI (**Figure 3F**). These consistent and notable age-associated trends among T2DM risk and obesity reinforce conclusions from previous literature regarding the importance of age stratification in NHPI populations (Uchima et al., 2019).

### 3.2 Gut microbial population dynamics among T2DM risk factors

**Figure 4:**
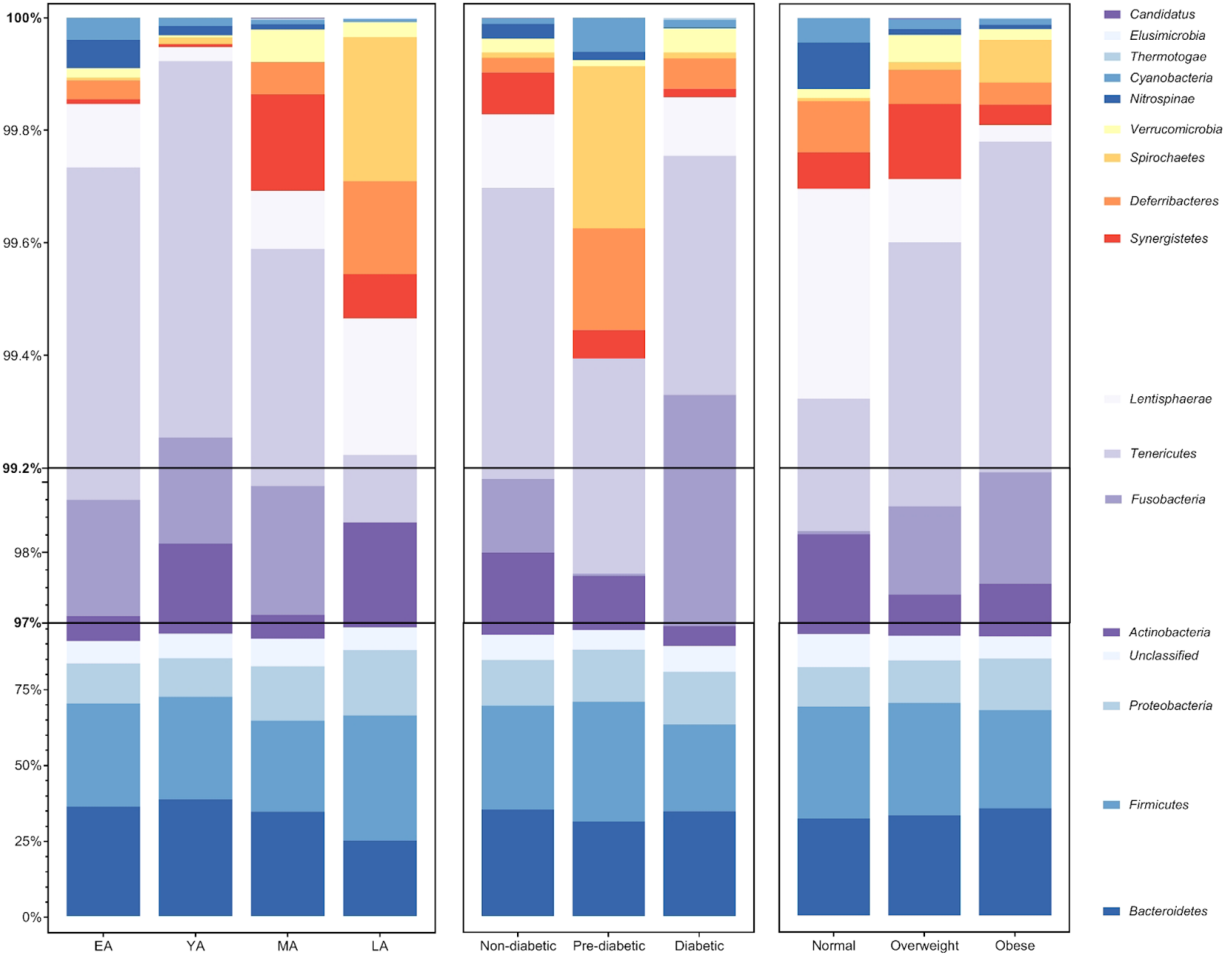
Average relative abundance of gut bacterial phyla across age groups, T2DM categories, and BMI categories. Bacterial phyla *Bacteroidetes, Firmicutes, Proteobacteria*, and *Actinobacteria* (among unclassified bacteria) largely compose an average of roughly 97% of the gut bacterial population in each group. The remaining 3% of gut bacteria are largely composed of members belonging to 12 additionally identified phyla: *Fusobacteria, Tenericutes, Lentisphaerae, Synergistetes, Deferribacteres, Spirochaetes, Verrucomicrobia, Nitrospinae, Cyanobacteria, Thermotogae, Elusimicrobia*, and *Candidatus Thermoplasmatota*.

Relationships between gut bacterial taxa and age, T2DM risk, and BMI are summarized in **Table 2**. Taxa described as butyrate-producers in previous literature are denoted by an asterisk (Louis and Flint, 2009; Vital et al., 2014; van den Berg et al., 2021; Das et al., 2022; Jaagura et al., 2022; Lee et al., 2022; Sasaki et al., 2022). As an exploratory measure, we chose to consider gut bacterial relationships with biometric data at statistical significance of *P<0.10*. The average relative abundance of identified gut bacterial phyla across age groups, T2DM categories, and BMI categories is summarized in **Figure 4**.

**Table 2:** Intergroup comparisons and correlation analyses for gut bacterial population distribution across age groups, T2DM categories, and BMI categories.

	Intergroup comparisons (P-values)^a^			Correlation analyses^b^					
				Age		A1c		BMI	
	Age groups	T2DM categories	BMI categories	R	P	R	P^b^	R	P^b^
**Phylum relative abundance**									
Actinobacteria*	**0.007**	**0.069**	0.823	**-0.26**	**0.002**	-0.09	0.310	-0.06	0.462
Bacteroidetes*	**<0.001**	0.376	0.823	**-0.25**	**0.003**	**-0.20**	**0.020**	**0.15**	**0.086**
* Cyanobacteria*	0.150	**0.016**	0.634	0.06	0.496	**0.17**	**0.051**	-0.10	0.234
Deferribacteres*	**<0.001**	**<0.001**	0.289	**0.30**	**<0.001**	**0.18**	**0.032**	-0.05	0.535
* Elusimicrobia*	0.419	0.373	0.823	0.13	0.141	0.11	0.219	0.03	0.765
Firmicutes*	**0.050**	**0.059**	0.598	0.07	0.403	0.06	0.455	**-0.16**	**0.070**
Fusobacteria*	0.279	**0.095**	0.469	-0.04	0.667	0.07	0.440	**0.20**	**0.019**
* Lentisphaerae*	0.904	**0.084**	0.535	0.03	0.724	**-0.15**	**0.087**	**-0.23**	**0.008**
* Nitrospinae*	0.458	0.981	0.823	0.01	0.926	-0.05	0.604	0.02	0.845
* Proteobacteria*	**0.008**	0.599	0.823	**0.28**	**<0.001**	0.09	0.300	0.07	0.450
Spirochaetes*	**0.011**	0.307	0.823	0.14	0.110	0.09	0.310	-0.08	0.365
Synergistetes*	0.113	0.867	0.535	0.10	0.258	0.01	0.959	-0.12	0.158
Tenericutes*	0.152	**0.023**	0.240	**0.14**	**0.090**	0.13	0.120	-**0.15**	**0.071**
Thermotogae*	0.418	**0.062**	0.823	0.06	0.468	0.12	0.149	0.03	0.699
* Verrucomicrobia*	0.142	0.428	0.919	0.11	0.211	0.08	0.382	0.03	0.753
**Genus relative abundance**									
Bifidobacterium*	**0.003**	0.262	0.484	**-0.33**	**<0.001**	**-0.20**	**0.019**	**-0.17**	**0.046**
Faecalibacterium*	0.484	**0.028**	**0.030**	-0.11	0.223	**-0.23**	**0.007**	**-0.15**	**0.077**
* Lactococcus*	0.685	**0.003**	0.828	-0.03	0.732	**0.18**	**0.034**	-0.05	0.574
* Mucispirillum*	**<0.001**	**<0.001**	0.273	**0.30**	**<0.001**	**0.18**	**0.032**	-0.06	0.517
* Prevotella*	0.111	**0.006**	0.217	**-0.12**	**0.043**	**-0.14**	**0.101**	0.02	0.849
Ruminococcus*	0.781	**0.003**	0.346	-0.11	0.202	-0.03	0.713	0.10	0.266
* Serratia*	0.786	**0.021**	0.164	0.08	0.376	-0.01	0.911	0.03	0.758
* Shigella*	**0.023**	**0.004**	0.543	**0.18**	**0.032**	**0.17**	**0.041**	0.05	0.566
Streptococcus*	0.306	**0.008**	0.697	<0.001	0.993	0.02	0.809	-0.01	0.950
**α-Diversity**^c^									
Chao-1	0.200	**0.062**	**0.105**	0.01	0.932	-0.01	0.946	0.13	0.143
Shannon	0.439	**0.028**	**0.078**	0.02	0.868	0.04	0.678	**0.18**	**0.053**
Simpson	0.289	**0.026**	**0.056**	0.02	0.828	0.07	0.427	**0.17**	**0.060**

Bold P-values indicate statistical significance at α=0.1.

a Kruskal-Wallis ANOVA.

b Spearman rank-order correlation coefficient.

c Taxonomic diversity at the genus-level.

*Butyrate-producing taxa.

**Figure 5:**
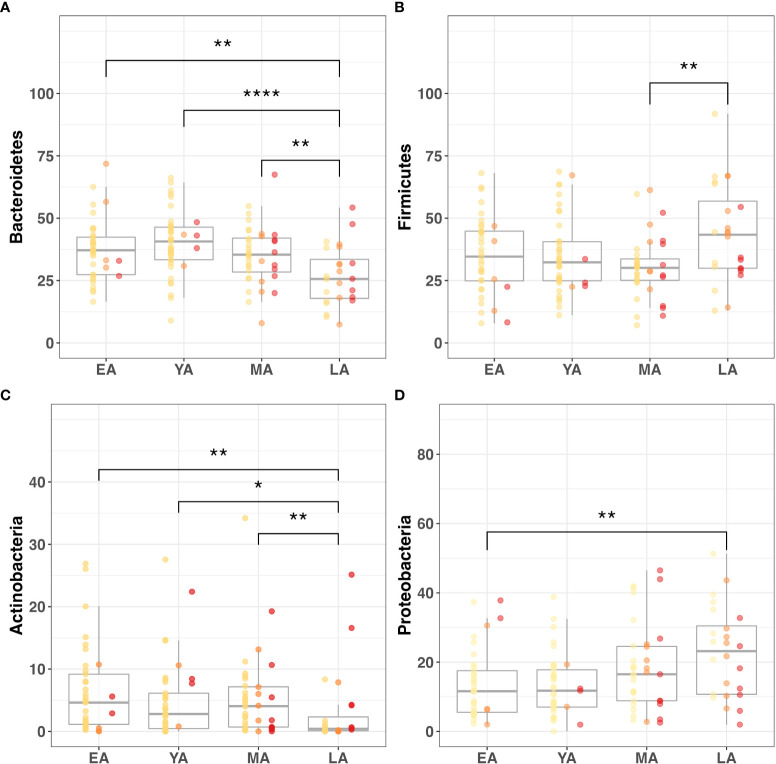
Gut microbiome composition dynamics upon age stratification. Graphical representation of significantly different relative abundance of four major gut bacterial phyla across age groups. Color-coded indications of individual data points categorized by T2DM risk: non-diabetic (yellow), pre-diabetic (orange), and diabetic (red). Visualized phyla include **(A)** *Bacteroidetes*, **(B)** *Firmicutes*, **(C)** *Actinobacteria*, **(D)** Proteobacteria. Statistical significance denoted in figures above using asterisks as follows: * (P<0.05); ** (P<0.01); **** (P<0.0001).

Age group comparisons for the relative abundance of four major gut bacterial phyla are illustrated in **Figure 5**. The relative abundance of *Bacteroidetes (* **Figure 5A** **;** *P<0.001), Firmicutes (* **Figure 5B** **;** *P=0.050), Actinobacteria* (**Figure 5C** **;** *P=0.007)*, *Proteobacteria* (**Figure 5D** **;** *P=0.008), Deferribacteres (P<0.001)*, and *Spirochaetes (P=0.011)* significantly differed across age groups. Among these relationships, significant correlations were observed between age and *Actinobacteria (R=-0.26, P=0.002)*, *Bacteroidetes (R=-0.25; P=0.003)*, *Deferribacteres (R=0.30; P<0.001)*, and *Proteobacteria (R=0.28; P<0.001).* It should be noted that *Proteobacteria* uniquely exhibited a significant correlation with age and differential relative abundance across age groups without demonstrating strong direct associations with indicators of T2DM risk or BMI.

Age also demonstrated a marginally positive correlation with the relative abundance of *Tenericutes (R=0.14; P=0.090)*. While this relationship with age uniquely accompanied a negative correlation with BMI (*R=-0.15; P=0.077)*, intergroup variance for relative *Tenericutes* abundance was nonsignificant for age groups *(P=0.152*) and BMI categories *(P=0.240*). Interestingly, its relative abundance significantly varied across T2DM categories *(P=0.023)* while no such relationship was observed with nominal A1c values (*P=0.120)*.

The relative abundance of *Actinobacteria (P=0.069), Cyanobacteria (P=0.016), Deferribacteres (P<0.001), Firmicutes (P=0.059), Fusobacteria (P=0.095), Lentisphaerae (P=0.084), Tenericutes (P=0.023)* and *Thermotogae (P=0.062)* significantly differed across T2DM categories. Among these trends, A1c significantly correlated with *Cyanobacteria (R=0.17; P=0.051) and Deferribacteres (R=0.18; P=0.032)*, possibly suggesting an influential or bidirectional relationship between glucose homeostasis and gut bacterial members of these two phyla. Additionally, A1c correlated negatively with the relative abundance of Lentisphaerae *(R=-0.15; P=0.087)*. This phylum had a notably parallel relationship with BMI *(R=-0.23; P=0.008)*, exhibiting consistently negative directionality between increasing glycemia and obesity. However, implications of this integrated functional relevance coincide with nonsignificant variance in relative Lentisphaerae abundance across BMI categories *(P=0.535)*.

Relative *Bacteroidetes* abundance similarly exhibited overlapping correlations with A1c and BMI. Relative *Bacteroidetes* abundance was observed to simultaneously decrease with glycemia (*R=-0.20; P=0.020*) and increase with obesity (*R=0.15; P=0.086*). Although relative abundance of this phylum did not significantly vary across T2DM (*P=0.376*) and BMI (*P=0.823*) categories, it uniquely correlated with age, A1c, and BMI.

Genera that were differentially abundant across age groups included *Bifidobacterium (P=0.003)*, *Mucispirillum (P<0.001)* and *Shigella (P=0.023)*. Of these genera, *Bifidobacterium (R=-0.33; P<0.001)* correlated negatively with age, while *Mucispirillum (R=0.301; P<0.001)* and *Shigella (R=0.182; P=0.032)* correlated positively. While it demonstrated no statistical difference across age groups, the relative abundance of *Prevotella* also correlated negatively with age *(R=-0.17; P=0.043)*. Notably, each of these age-related genera demonstrated parallel correlations to A1c.

Genera exhibiting significant correlations to A1c included *Bifidobacterium (P=-0.20; 0.019), Faecalibacterium (R=-0.23; P=0.007), Lactococcus (R=0.18; P=0.034), Mucispirillum (R=0.18; P=0.032), Prevotella (R=-0.14; P=0.101), and Shigella (R=0.17; P=0.041)*. The relative abundance of all genera discussed for the purpose of this study distributed differentially across T2DM groups except one, Bifidobacterium, the only genus to simultaneously exhibit negative correlations among age, A1c, and BMI *(R=-0.017; P=0.046)*. The only other observed genus to simultaneously correlate with A1c and BMI in relative abundance was *Faecalibacterium (R=-0.15; P=0.077)*, which was unique in its differential distribution across both T2DM (*P=0.028*) and BMI (*P=0.030*) categories. Notably, *Faecalibacterium* was the only taxon among reported phyla and genera with significant differences in abundance across BMI categories. While seemingly specific interactions with T2DM risk were observed for *Lactococcus (P=0.003), Ruminococcus (P=0.003), Serratia (P=0.021)*, and *Streptococcus (P=0.008), Lactococcus* uniquely exhibited a corresponding correlation with A1c levels (*R=0.18; P=0.034*).

**Figure 6:**
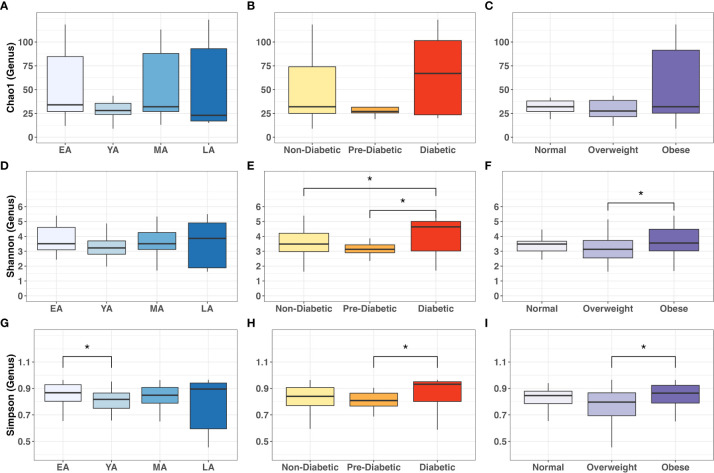
Intergroup comparisons for gut bacterial α-diversity at the genus level across age groups, T2DM categories, and BMI categories. Mean comparisons were performed across **(A, D, G)** age groups; **(B, E, H)** T2DM categories, and **(C, F, I)** BMI categories for Chao1, Shannon and Simpson indices, respectively. Statistical significance denoted: * (*P*<0.05).

We then applied Dunn’s *post-hoc* test for multiple-comparisons to the genus-level α-diversity indices across age, T2DM risk and BMI categories, summarized in **Figure 6**. Chao-1 did not differ between age, T2DM risk and BMI groups **(** **Figures 6A-C** **)**. Shannon index values did not significantly vary between age groups **(** **Figure 6D**) but were significantly higher in diabetics compared to non-diabetics *(P=0.042*) and pre-diabetics *(P=0.018)* **(** **Figure 6E** **)**, and similarly higher in obese individuals compared to overweight individuals *(P=0.031)* **(** **Figure 6F** **)**. Simpson index values demonstrated intergroup significance in all three categorical variables, EA and YA age groups *(P=0.042)* **(** **Figure 6G** **)**, pre-diabetics and diabetics *(P=0.018)* and between overweight and obese *(P=0.021)* participants.

### 3.3 *BUK-*mediated butyrate metabolism

**Figure 7:**
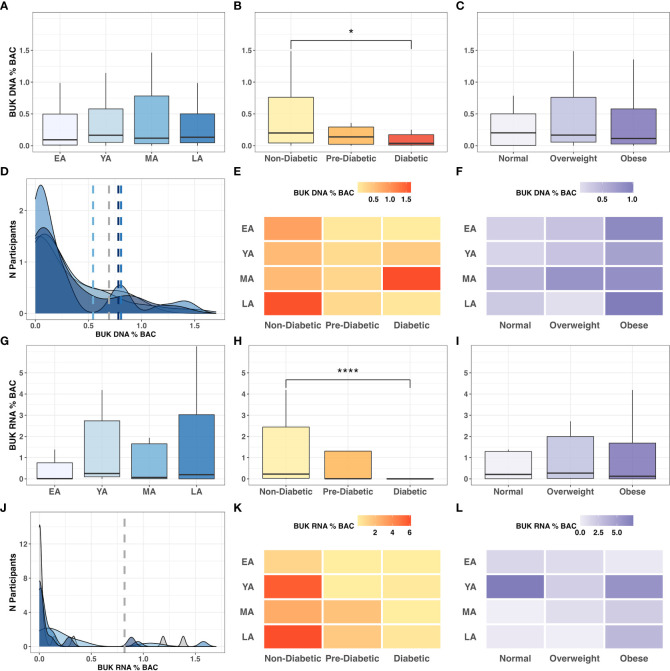
*BUK* gene abundance and expression relative to BAC across age, T2DM and BMI categories. Intergroup comparison of relative *BUK* gene abundance across **(A)** age groups *(P=0.802*), **(B)** T2DM categories (*P=0.032)*, and **(C)** BMI categories (*P=0.736*). Frequency distribution of participants by age groups among binned *BUK* DNA values is summarized by a color-coded density plot fitted over a histogram **(D)**. Heatmaps indicating the range of relative *BUK* DNA distributed across age groups and **(E)** T2DM categories and **(F)** BMI categories. Intergroup comparison of relative *BUK* RNA quantitation across **(G)** age groups (*P=0.151*), **(H)** T2DM categories (*P<0.001)*. and **(I)** BMI categories (*P=0.636*). Frequency distribution of participants by age groups among binned *BUK* RNA values is summarized by a color-coded density plot fitted over a histogram **(J)**. Heatmaps indicating the range of relative *BUK* RNA distributed across age groups and **(K)** T2DM categories and **(L)** BMI categories. Statistical significance denoted in figures above using asterisks as follows: * (*P*<0.05); **** (*P*<0.0001).

To explore metabolic mechanisms underlying functional interactions between microflora and health outcomes in NHPIs, we quantified gut bacterial capacity for *BUK*-mediated butyrate metabolism by measuring the abundance of *BUK* DNA and RNA relative to total bacterial load per stool sample. Observed trends in relative levels of *BUK* abundance among age groups, T2DM categories, and BMI categories are summarized in **Figure 7**. While the relative abundance of *BUK* DNA did not significantly vary between age groups (**Figure 7A** **),** it significantly decreased across successive T2DM categories (**Figure 7B** **;** *P=0.032)* with a notable reduction in diabetics compared to non-diabetics (*P<0.05).* This relationship with host physiology did not extend to differential abundance across BMI categories (**Figure 7C** **).**

Without statistical indicators of direct interactions among *BUK* gene abundance and biometric data, we investigated these relationships more broadly. Variance in *BUK* levels between age groups was visualized using a fitted population density histogram (**Figure 7D** **).** Relatively low *BUK* gene abundance was observed most frequently in MA individuals compared to other age groups. While LA individuals exhibited low *BUK* DNA, the levels of which were similar to those of younger individuals (EA-YA), notable peaks were observed among LA individuals with moderate to high levels of *BUK* DNA. While exhibiting significant differences across T2DM categories, relative *BUK* DNA abundance did not show a clearly discernible pattern across intersecting age groups and T2DM categories (**Figure 7E** **).** Aside from an anomalously high *BUK* DNA abundance in diabetic MA individuals, increased *BUK* gene abundance seemed to tend toward non-diabetics, while demonstrating a more ambiguous distribution across age groups. This ambiguous relationship with age was consistent with that observed for *BUK* DNA levels across age groups and BMI categories, although elevated *BUK* gene abundance tended toward individuals with high BMI values (**Figure 7F** **).**

Compared to patterns observed for *BUK* DNA, low *BUK* RNA was observed at a notably higher frequencies across all age groups, with fewer observations of increased levels (**Figure 7J** **).** The relative abundance of *BUK* RNA distributed across age groups and T2DM categories revealed higher levels among non-diabetic individuals and showed an ambiguous distribution across age (**Figure 7K** **).** This ambiguity with age extended to age groups and BMI categories, with higher values observed among obese individuals, except for an anomalously high relative *BUK* RNA abundance in YA individuals with normal BMI (**Figure 7L** **)**.

To better understand this ambiguity and as previous literature has reported strong associations between dietary habits and gut bacterial butyrate metabolism (Das et al., 2022), we investigated whether the relationship between the gut microbiome and host health outcomes may be modulated by fiber consumption, primarily from vegetable sources, as a potential avenue for T2DM risk reduction. To this end, we leveraged known metrics of dietary quality and developed a new dietary score based on self-reported data with a focus on vegetable sources of dietary fiber intake called “VEG^2^” defined below.

### 3.4 VEG^2^ as a measure of dietary quality in NHPIs

The Multi-Ethnic Cohort (MEC) study based at the University of Hawaii Cancer Center included data collected from 215,000 residents of Hawaii and Los Angeles through a 26-page questionnaire (including questions regarding behavioral risk factors and medical history) with follow-up questionnaires administered in five-year intervals (University of Hawaii). Biological samples were collected from more than 70,000 MEC members in 2001-2005. One MEC sub-study presented NHPI quintile distribution data for a number of conventionally accepted dietary quality indices (DQIs): the Approaches to Stop Hypertension (DASH) and the Healthy Eating Index (HEI-2010) (Jacobs et al., 2015). We accessed this data and obtained a summary of dietary index score quintile distribution data among NHPI men and women (provided in supplemental materials). Quintile distribution was based on dietary index range, from Q1 (lowest scores) to Q5 (highest scores). Participant scores were treated separately for men and women to control for sex-related differences.

For the purpose of our study, a modified DQI, VEG^2^, was formulated to assess fiber supplementation *via* vegetable consumption in NHPI populations. Survey questions used to collect dietary data were designed to quantify individuals’ variety and frequency of vegetable consumption relative to that of meats, fish, refined sugars, processed carbohydrates, and more using a point-value system assigned to diet-related survey responses. An overview of questions used for vegetable-related dietary data collection and their corresponding point-value assignment is provided in **Table S3**, followed by the formulas used to calculate the VEG^2^ metric. Chi-squared tests for independence comparing quintile distribution of NHPIs between DASH, HEI-2010, and VEG^2^ (**Table S4**) indices were nonsignificant (**Table S5**). Thus, we propose that the VEG^2^ DQI is functionally similar to established metrics in providing a general overview of self-reported dietary quality in NHPIs, with a focus on dietary sources of fiber intake.

**Figure 8:**
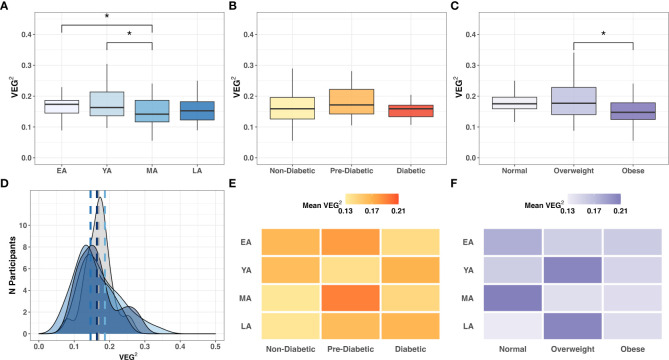
Intergroup comparison of VEG^2^ across **(A)** age groups *(P=0.064*); **(B)** T2DM categories (*P=0.396*); and **(C)** BMI categories (*P=0.005)*. Frequency distribution of participants by age groups among binned VEG^2^ scores is summarized by a color-coded density plot fitted over a histogram **(D)** Heatmaps indicating the range of VEG^2^ scores distributed across age groups and **(E)** T2DM categories or **(F)** BMI categories. Statistical significance denoted: * (*P*<0.05).

In our cohort study, VEG^2^ correlated negatively with age (**Table 3** **;** *R=-0.17; P=0.051*), differed across age groups (**Figure 8A** **;** *P<0.005)*, and did not significantly differ across T2DM categories **(** **Figure 8B** **)**. VEG^2^ also correlated negatively with BMI (*R=-0.25; P=0.004*) and significantly varied across BMI categories (**Figure 8C**; *P=0.005*). Direct associations were not observed between VEG^2^ and *BUK* gene abundance *(R=-0.05; P=0.573)* or expression *(R=0.02; P=0.804)*. The distribution of VEG2 score frequency clustered similarly among YA, MA, and LA age groups, while that of EA tended toward a higher median with higher frequency (**Figures 8D**). Mean VEG^2^ scores appeared to be dependent on BMI per age group, while the relationship was less clear in the context of T2DM risk (**Figures 8E, F**).

**Table 3:** Comparisons between biometrics, microbial abundances, VEG^2^ scores and *BUK* levels.

VEG^2^		*BUK* DNA		*BUK* RNA	
	R	P^a^	R	P^a^	R	P^a^
**Age**	-0.17	0.051	0.046	0.631	0.02	0.833
**A1c**	0.03	0.705	-0.17	0.071	-0.33	0.003
**BMI**	-0.25	0.004	-0.03	0.783	-0.08	0.479
**VEG^2^**	–	–	-0.05	0.573	0.02	0.840
**Phylum Relative Abundance**						
* Verrucomicrobia*	-0.20	0.018	-0.08	0.379	-0.18	0.114
**Genus Relative Abundance**						
* Mannheimia*	0.26	0.002	0.09	0.340	-0.13	0.261
* Blautia*	-0.22	0.011	-0.07	0.463	-0.09	0.450
* Senegalimassilia*	-0.20	0.019	-0.02	0.807	-0.17	0.123
* Haemophilus*	0.19	0.025	0.19	0.045	0.09	0.434
* Subdoligranulum*	-0.19	0.026	0.02	0.799	0.00	0.993
* Victivallis*	-0.18	0.035	-0.03	0.776	0.04	0.750
* Photobacterium*	0.18	0.036	0.13	0.182	-0.09	0.404
* Ruminococcus 2*	-0.17	0.044	0.03	0.741	0.02	0.886
* Catenibacterium*	-0.17	0.050	-0.09	0.323	0.09	0.401
* Akkermansia*	-0.15	0.085	-0.24	0.012	-0.22	0.049
* Klebsiella*	-0.13	0.121	0.25	0.007	-0.05	0.645
* Enterococcus*	-0.05	0.543	0.01	0.917	0.26	0.021
* Lactobacillus*	0.05	0.545	0.09	0.372	0.30	0.006
* Bacteroides*	0.01	0.950	0.02	0.807	-0.28	0.011
**Species Relative Abundance**						
* Mannheimia varigena*	0.26	0.002	0.11	0.238	-0.12	0.273
* Collinsella tanakaei*	0.25	0.003	0.06	0.534	-0.04	0.741
* Prevotella stercorea*	-0.21	0.012	-0.02	0.847	0.08	0.499
* Holdemania massiliensis*	0.21	0.014	0.07	0.442	0.00	1.000
* Lactobacillus salivarius*	-0.21	0.015	-0.02	0.826	0.11	0.349
* Dorea formicigenerans*	-0.20	0.019	0.09	0.358	0.11	0.325
* Blautia glucerasea*	-0.20	0.022	0.02	0.849	0.12	0.297
* Collinsella aerofaciens*	-0.20	0.022	-0.08	0.379	0.00	0.976
* Haemophilus parainfluenzae*	0.19	0.023	0.19	0.040	0.14	0.225
* Ruminococcus torques*	-0.19	0.024	-0.06	0.505	-0.15	0.195
* Victivallis vadensis*	-0.18	0.033	-0.03	0.776	0.04	0.750
* Streptococcus peroris*	-0.18	0.034	0.00	0.987	0.24	0.030
* Slackia isoflavoniconvertens*	-0.18	0.039	0.05	0.632	0.01	0.964
* Eubacterium hallii*	-0.18	0.039	-0.09	0.336	-0.03	0.774
* Parabacteroides johnsonii*	0.18	0.040	0.05	0.605	0.12	0.288
* Clostridium bartlettii*	0.18	0.040	0.05	0.605	0.12	0.288
* Akkermansia muciniphila*	-0.15	0.071	-0.25	0.009	-0.22	0.053
* Helicobacter hepaticus*	0.13	0.129	0.06	0.528	-0.25	0.027
* Streptococcus australis*	-0.12	0.174	0.01	0.886	0.24	0.031
* Lactobacillus ruminis*	-0.11	0.210	0.04	0.697	0.25	0.023
* Clostridium paraputrificum*	0.10	0.231	0.13	0.172	-0.25	0.024
* Streptococcus salivarius*	-0.10	0.259	-0.04	0.674	0.24	0.031
* Bacteroides stercoris*	-0.08	0.377	0.00	0.990	-0.23	0.040
* Clostridium lavalense*	0.07	0.399	-0.23	0.016	-0.08	0.494
* Ruminococcus callidus*	-0.07	0.409	0.01	0.941	0.23	0.041
* Oscillibacter* sp.	0.06	0.472	-0.19	0.050	0.07	0.509
* Bifidobacterium angulatum*	-0.06	0.519	-0.04	0.688	0.24	0.030
* Serratia rubidaea*	-0.06	0.523	0.22	0.021	-0.01	0.907
* Ruminococcus* sp.	-0.03	0.690	-0.21	0.023	-0.15	0.177
* Ruminococcus gnavus 2*	0.03	0.699	0.15	0.111	-0.26	0.019
* Helicobacter ganmani*	0.02	0.775	0.15	0.123	0.26	0.018
* Sutterella* sp.	-0.01	0.904	-0.12	0.198	0.27	0.013
* Desulfovibrio d168*	-0.01	0.905	0.20	0.032	0.21	0.065
* Veillonella rogosae*	-0.01	0.934	0.21	0.024	-0.04	0.702
* Parabacteroides goldsteinii*	0.01	0.939	-0.03	0.773	0.25	0.026

a Spearman rank-order correlation coefficient.

A1c negatively correlated with *BUK* DNA *(R=-0.17; P=0.071)* and RNA *(R=-0.33; P=0.031)*, suggesting a functional distinction between dietary and biometric interactions with gut bacteria. Relative *Verrucomicrobia* abundance uniquely correlated with VEG^2^ (*R=-0.20; P=0.018*) without exhibiting direct relationships between age, T2DM risk, or BMI (**Table 2** **)**. *Haemophilus* was the only genus whose abundance significantly correlated with both VEG^2^ *(R=0.19; P=0.025)* and BUK gene abundance *(R=0.19; P=0.045)*. *Akkermansia* was the only genus that demonstrated significant negative correlations with BUK gene abundance *(R=-0.24; P=0.012)* and expression *(R=-0.22; P=0.049)*. Genera whose levels exhibited a positive correlation with *BUK* gene abundance were *Mannheimia (R=0.26; P=0.002) and Photobacterium (R=0.18; P=0.036)*. Negative correlations were observed between *Blautia (R=-0.22, P=0.011), Subdoligranulum (R=-0.19; P=0.026)*, *Victivallis (R=-0.18; P=0.035), Ruminococcus 2 (R=-0.17; P=0.044)* and *Catenibacterium (R=-0.17; P=0.050)*. Genera that exhibited a positive correlation with *BUK* gene expression were *Enterococcus (R=0.26; P=0.021)* and *Lactobacillus (R=0.30; P=0.006)*, with *Bacteroides (R=-0.28; P=0.011)* showing a negative relationship. Without indication of direct relationships with age or BMI **(** **Table 3** **)**, the relative levels of *BUK* DNA and *BUK* RNA demonstrated marginal (*R=-0.17; P=0.071*) and strong (*R=-0.33; P=0.031)* negative correlations with A1c, respectively. Additional data regarding dietary associations are summarized in **Table S6**.

## 4 Discussion

Due to the underrepresentation of NHPIs in biomedical research studies, data from our NHPI cohort provides an example of potentially race/ethnic-specific relationships that would have not been previously recognized. Exploratory analyses of T2DM risk factors in our NHPI cohort revealed dynamic biometric and sociodemographic patterns, some of which were unexpected based on studies in other race/ethnic groups. Although A1c and BMI are commonly associated with each other, the positive correlation observed between the two (*R=0.18; P=0.030)* was not as strong as that of A1c and age (*R=0.39; P<0.001).*

The lack of overlap between A1c and BMI as predictors of T2DM-related health outcomes implicate the two metrics as inequivalent indicators of cardiometabolic disease risk. While conventional associations between obesity and T2DM risk have limited applicability to NHPI populations, age may be more effective as a predictor of cardiometabolic pathophysiology. Further discrepancies between documented trends among gut microbial influence on host physiology and those observed in our cohort emphasize unforeseen functional independence between BMI and T2DM risk in NHPIs. Significant differences in relative phylum abundance were not observed across BMI categories, contrasting with direct correlations observed between BMI values and five phyla (**Table 2** **)**. This discrepancy may suggest that interactions between gut bacteria and host adiposity are functionally indirect. Further mediation analyses are required to assess indirect effects of these interactions.

*Bifidobacterium* is the most conventionally reported to be an antagonist against physiological determinants of T2DM-related complications (Ma et al., 2021). The negative correlation between *Bifidobacterium* and age (*R=-0.32; P<0.001)*, consistent with reports from existing literature, was further supported by negative correlations with A1c (**Table 2** **;** *R=-0.20; P=0.019)* and BMI (*R=-0.17; P=0.046)*. While exploratory statistics may implicate certain bacteria as partial determinants of glucose homeostasis or adiposity, their effects on host physiology are neither direct nor isolated, as the population dynamics and metabolic activity observed in gut bacteria are interdependent on one another.

Existing literature led us to expect an inverse relationship between gut bacterial α-diversity and T2DM risk. Cross-sectional studies have reported negative associations between α-diversity and insulin resistance in the majority of race/ethnic groups examined (Chen et al., 2021), suggesting a protective relationship between a diverse bacterial population and T2DM pathophysiology. While we observed contradictory results in our NHPI-cohort, further investigation, including increasing representation of NHPIs across diverse communities, is necessary to better understand these relationships.

Among the gut microbial features under our investigation in this study, we did not observe a direct relationship of relative *BUK* DNA abundance with age or BMI **(** **Table 3** **).** However, a positive relationship with A1c was observed *(R=-0.17; P=0.071)*. The same trends were observed with *BUK* RNA levels, as a significant relationship was only observed with A1c *(R=-0.33; P=0.031)*. These results suggest that reduced *BUK* activity in the gut corresponds with an increase in A1c and T2DM risk, likely through glucose homeostatic pathways.

Meta-analyses investigating the relationship between DQIs and health outcomes have found an inverse association between both DASH and HEI scores with the risk of incidence or mortality from cardiovascular disease and T2DM (Jacobs et al., 2015; Schwingshackl and Hoffmann, 2015). While such systematic reviews provide insight into conventional DQI score applications, it is unclear whether these trends are generalizable to indigenous populations, in particular to NHPIs. We propose the VEG^2^ score as a modified DQI with increased accessibility for members of NHPI communities. Due to the negative correlation between vegetable intake scores and age, we are unable to conclude whether a characteristic shift in diet with age is as significant a contributor to the shift in gut microbial population dynamics as is age alone. We note, however, that this decreased VEG^2^ score with age associated with increased BMI and T2DM risk in our NHPI-enriched cohort, implicating dietary deficiencies, in particular fiber intake, as a potential contributor to obesity and diabetes. Given the strong yet contrasting associations with VEG^2^ and *BUK*, our data implicates *Haemophilus* and *Akkermansia*, among others, in playing a role in potentially mediating the effects of diet on metabolic health in this cohort.

Our observations of dynamic interactions among age, gut microbiome, and T2DM risk factors support previous literature emphasizing the necessity for age stratification in health disparities research involving NHPI populations. Discrepancies in conclusions of relationships between gut microbial indicators of health outcomes may likely arise from race/ethnic variability, emphasizing that findings in other populations may not be generalizable to NHPI and other minority populations underpresented in biomedical research. Gut microbial mediation of host physiology affects obesity and T2DM risk in NHPIs in ways not yet well understood. NHPI-specific microbial dynamics may contribute to the cardiometabolic health disparity experienced by the NHPI community *via* microbial pathways that may be unique to this group. Thus, bridging the gap in biomedical data disparity for NHPI populations may allow for the development of more robust, community-based strategies to effectively address specific pathways underlying disparities among NHPIs.

## Data availability statement

All data used for this project will be available de-identified when approved by the University of Hawaii Institutional Review Board upon reasonable request to the corresponding author. The data presented in the study are deposited in the figshare repository, accessible at: https://doi.org/10.6084/m9.figshare.21637547.v1.

## Ethics statement

The studies involving human participants were reviewed and approved by University of Hawaii Institutional Review Board (UH IRB) under protocol number 2019-00376. The participants provided their written informed consent to participate in this study.

## Author contributions

Funding for this study was obtained by AM, who also conceptualized, organized, and led the study. RW and BK performed experiments, conducted analysis of biometric data, and wrote the manuscript alongside AM. RP oversaw all experiments, provided edits to the manuscript, and conducted data analysis on NGS data. KP oversaw participant recruitment, data curation, and created the sociodemographic survey and VEG^2^ metric alongside RJ. LU, TM, RL, and DK performed experiments. All authors reviewed the manuscript and approved the final submission.

## Supplementary material

Supplementary Figure 1 Inclusion-exclusion criteria for cohort participant data from the time of enrollment to metagenomic sequencing analysis.

Supplementary Figure 2 Relative abundance of gut microbial phyla per individual across **(A)** age group, **(B)** T2DM category and **(C)** BMI category.
