# Supplementary figures and images for: Gut microbial indicators of metabolic health underlie age-related differences in obesity and diabetes risk among Native Hawaiians and Pacific Islanders
Source: Front Cell Infect Microbiol. 2022 Dec 21;12:1035641. doi: 10.3389/fcimb.2022.1035641 (PMC9812644; doi:10.3389/fcimb.2022.1035641)

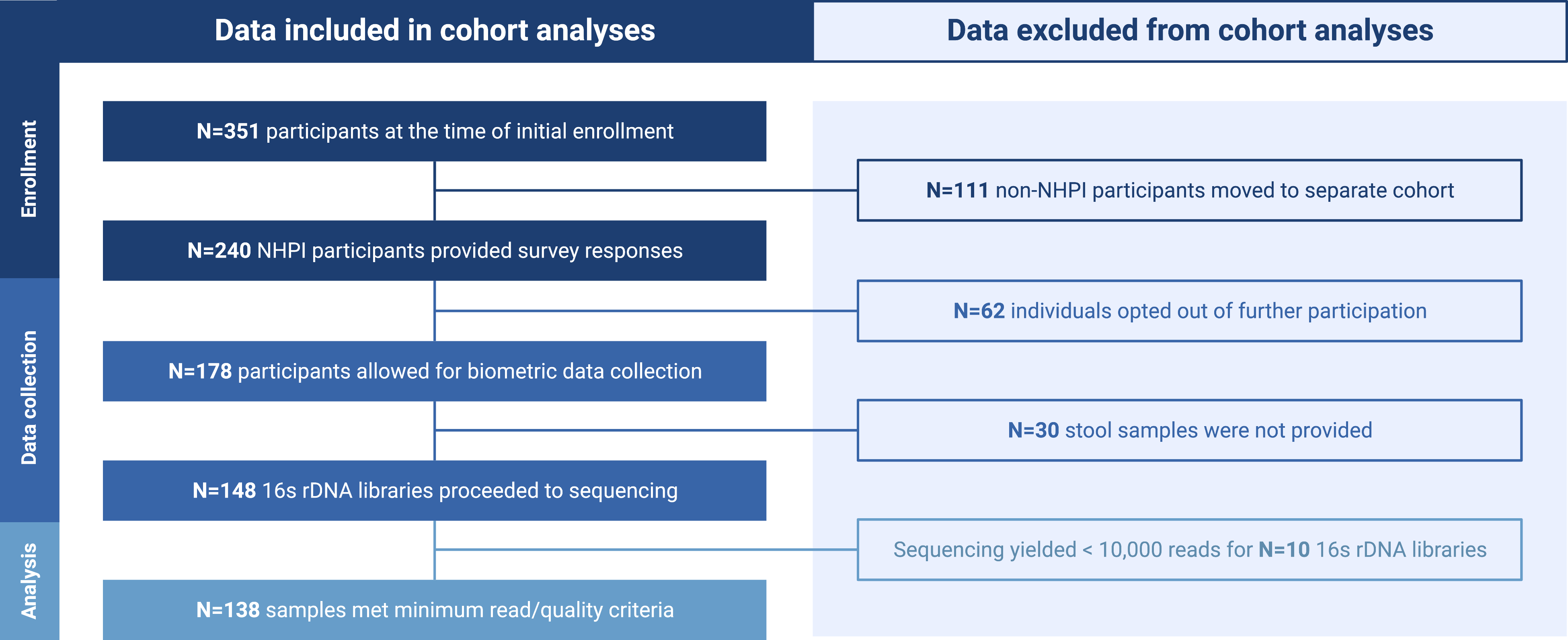

Supplement: Supplementary Figure 1 — Inclusion-exclusion criteria for cohort participant data from the time of enrollment to metagenomic sequencing analysis. [file Image_1.jpeg]

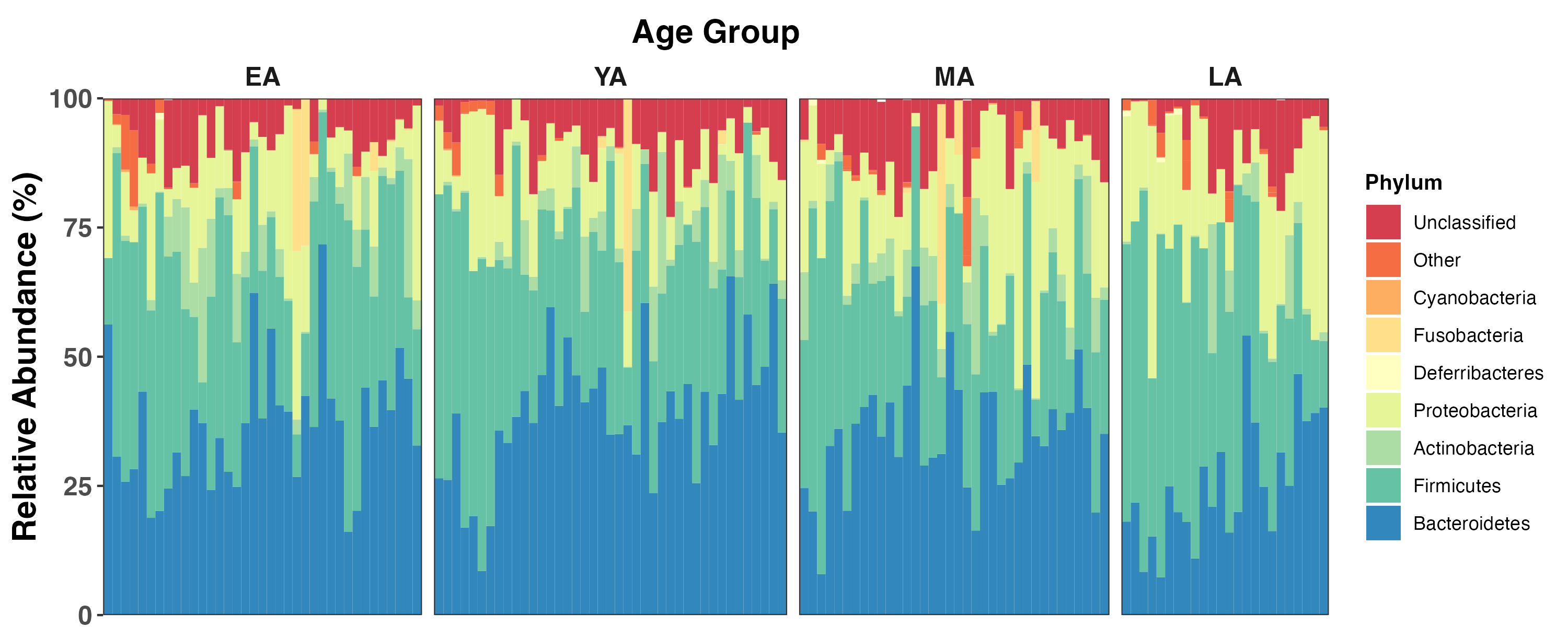

Supplement: Supplementary Figure 2 — Relative abundance of gut microbial phyla per individual across (A) age group, (B) T2DM category and (C) BMI category. [file Image_2.tiff]

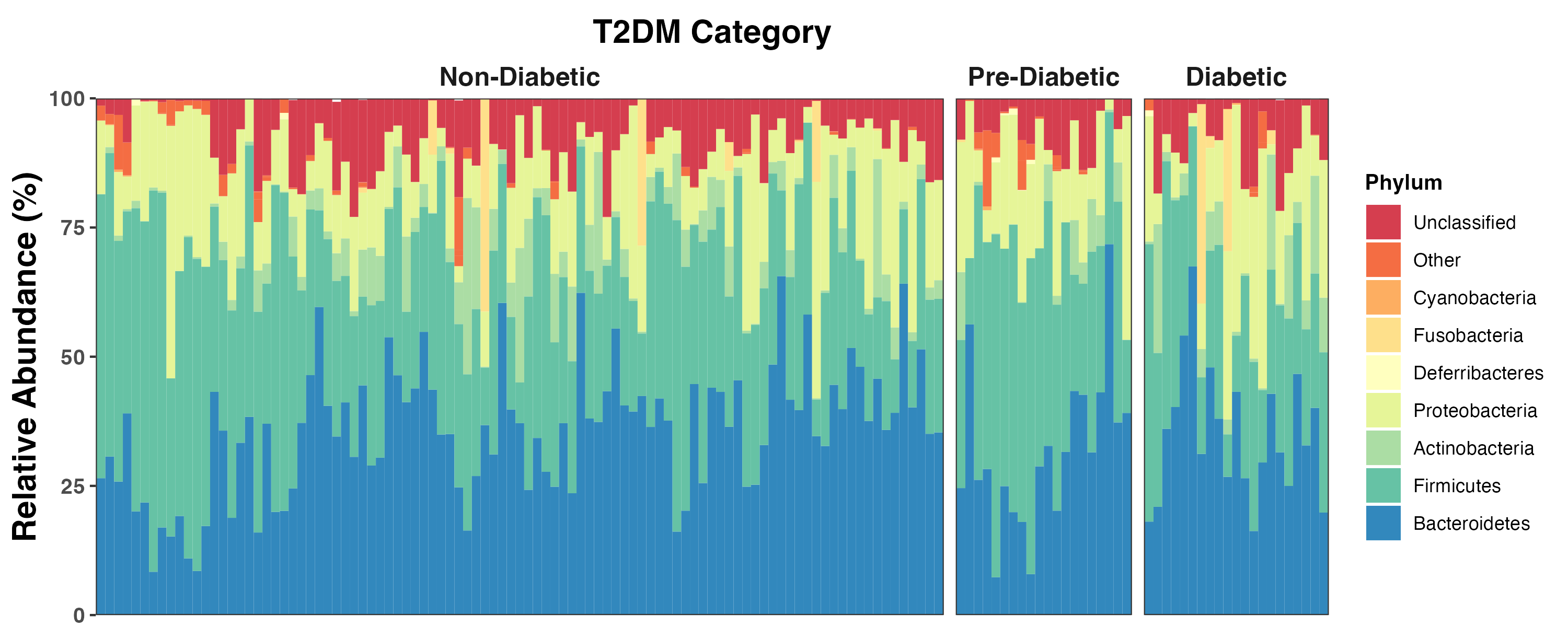

Supplement: Supplementary file 3 [file Image_3.tiff]

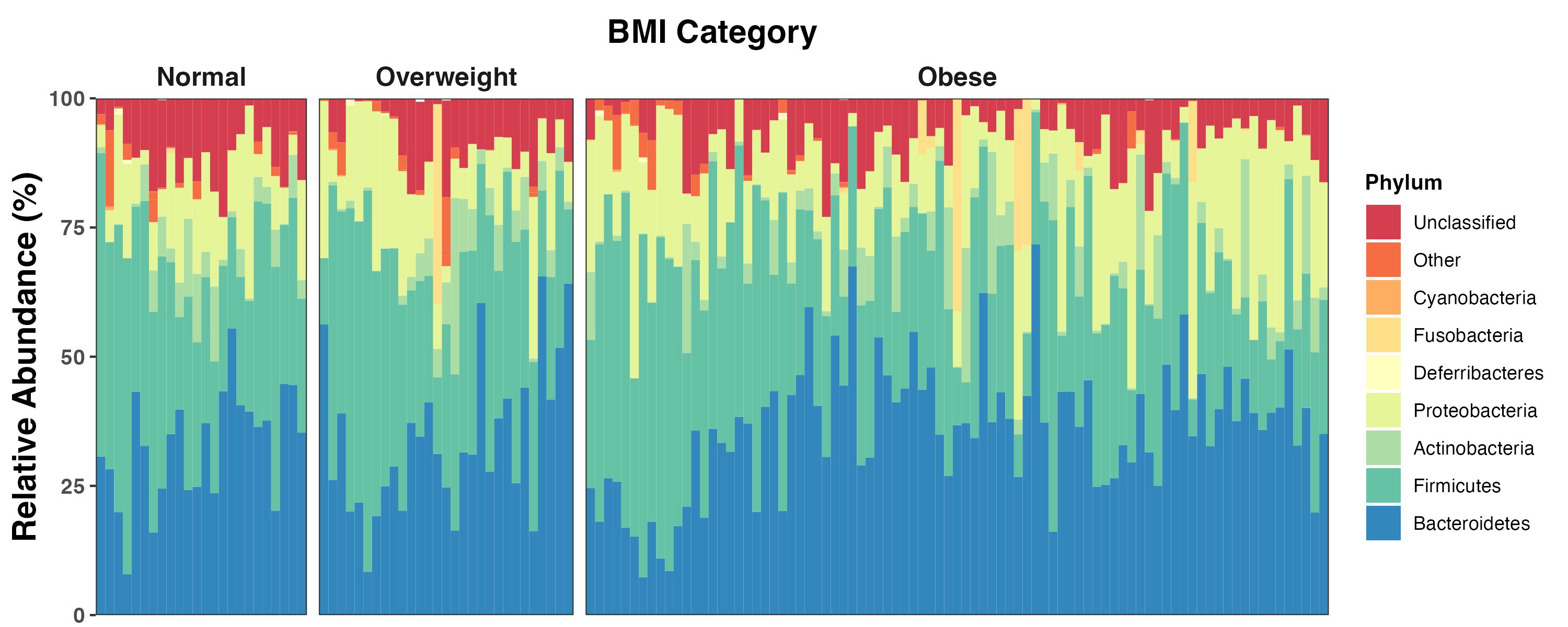

Supplement: Supplementary file 4 [file Image_4.tiff]
