# Supplementary material for: Gut microbial indicators of metabolic health underlie age-related differences in obesity and diabetes risk among Native Hawaiians and Pacific Islanders
Source: Front Cell Infect Microbiol. 2022 Dec 21;12:1035641. doi: 10.3389/fcimb.2022.1035641 (PMC9812644; doi:10.3389/fcimb.2022.1035641)
Supplement: Supplementary file 5 [file Table_1.docx]

**Supplemental Materials**

**Table S1**. 16s metagenomic sequencing quality for all samples analyzed (average and SEM shown).

|  | **Quality Control Metrics** | | | |
| --- | --- | --- | --- | --- |
|  | **Total Reads (thousands)** | **Mean Bases**  **(Mb)** | **Mean Read Length (bp)** | **Mean Q20 Bases**  **(%)** |
| **Overall (n=138)** | 333 ± 38.8 | 76 ± 8.963 | 225 ± 0.8174 | 88.24 ± 0.13 |

**Table S2**. Significant intergroup comparisons among age groups, T2DM categories, and BMI categories, accompanied by significant correlation analyses between the relative abundance of butyrate-producing bacteria (at the species level, grouped by genus) and age, A1c, BMI, and VEG^2^ scores. Highlighted cells indicate statistically significant values determined at *P<0.10.*

|  | **Intergroup comparisons^A^** | | |  |  | **Correlation analyses^B^** | | | | | | | | | | |
| --- | --- | --- | --- | --- | --- | --- | --- | --- | --- | --- | --- | --- | --- | --- | --- | --- |
|  |  |  |  |  |  | **Age** | |  | **A1c** | |  | **BMI** | |  | **VEG^2^ correlation** | |
|  | **Age** | **T2DM** | **BMI** |  |  | **R** | **P** |  | **R** | **P** |  | **R** | **P** |  | **R** | **P** |
| ***Bifidobacterium*** *(Actinobacteria)* | | | |  |  |  |  |  |  |  |  |  |  |  |  |  |
| *B. adolescentis* | 0.002 | 0.002 | 0.232 |  |  | -0.30 | <0.001 |  | -0.13 | 0.133 |  | -0.12 | 0.158 |  | 0.05 | 0.553 |
| *B. animalis* | 0.418 | 0.052 | 0.732 |  |  | 0.08 | 0.368 |  | 0.08 | 0.329 |  | 0.05 | 0.574 |  | 0.12 | 0.161 |
| *B. angulatum* | 0.053 | 0.064 | 0.186 |  |  | -0.23 | 0.007 |  | -0.17 | 0.041 |  | -0.14 | 0.109 |  | -0.06 | 0.519 |
| *B. bifidum* | 0.418 | 0.052 | 0.732 |  |  | 0.09 | 0.323 |  | 0.08 | 0.374 |  | 0.11 | 0.215 |  | -0.04 | 0.678 |
| *B. longum* | 0.003 | 0.059 | 0.273 |  |  | -0.23 | 0.008 |  | -0.28 | 0.001 |  | -0.15 | 0.076 |  | -0.08 | 0.355 |
| *B. pseudocatenulatum* | 0.489 | 0.604 | 0.048 |  |  | -0.05 | 0.528 |  | 0.05 | 0.559 |  | 0.12 | 0.16 |  | -0.06 | 0.475 |
| *B. pseudolongum* | 0.038 | 0.179 | 0.286 |  |  | 0.07 | 0.437 |  | 0.12 | 0.166 |  | -0.06 | 0.474 |  | 0.06 | 0.489 |
| *B. stercoris* | 0.114 | 0.432 | 0.047 |  |  | -0.19 | 0.028 |  | -0.13 | 0.125 |  | -0.11 | 0.202 |  | 0.06 | 0.507 |
| ***Faecalibacterium*** *(Firmicutes)* | | | |  |  |  |  |  |  |  |  |  |  |  |  |  |
| *F. prausnitzii* | 0.277 | 0.035 | 0.019 |  |  | -0.10 | 0.269 |  | -0.21 | 0.013 |  | -0.15 | 0.081 |  | -0.01 | 0.894 |
| ***Ruminococcus*** *(Firmicutes)* | | | |  |  |  |  |  |  |  |  |  |  |  |  |  |
| *R. faecis* | 0.046 | 0.034 | 0.155 |  |  | -0.21 | 0.014 |  | -0.15 | 0.073 |  | 0.06 | 0.48 |  | -0.14 | 0.093 |
| *R. flavefaciens* | 0.035 | 0.036 | 0.526 |  |  | 0.08 | 0.384 |  | -0.10 | 0.226 |  | -0.10 | 0.233 |  | -0.07 | 0.44 |
| *R. gauvreauii* | 0.539 | 0.001 | 0.594 |  |  | -0.01 | 0.887 |  | -0.07 | 0.408 |  | 0.00 | 0.972 |  | -0.15 | 0.09 |
| *R. gnavus* | 0.632 | 0.054 | 0.499 |  |  | -0.12 | 0.178 |  | 0.03 | 0.714 |  | -0.01 | 0.87 |  | -0.07 | 0.387 |
| *R. lactaris* | 0.186 | 0.197 | 0.018 |  |  | -0.10 | 0.228 |  | -0.09 | 0.278 |  | -0.14 | 0.102 |  | 0.01 | 0.91 |
| *R. torques* | 0.576 | 0.093 | 0.002 |  |  | -0.10 | 0.228 |  | 0.18 | 0.036 |  | 0.21 | 0.014 |  | -0.19 | 0.024 |
| ***Streptococcus*** *(Firmicutes)* | | | |  |  |  |  |  |  |  |  |  |  |  |  |  |
| *S. peroris* | 0.065 | 0.783 | 0.174 |  |  | 0.06 | 0.518 |  | 0.00 | 0.961 |  | 0.01 | 0.913 |  | -0.18 | 0.034 |
| *S. salivarius* | 0.073 | 0.779 | 0.304 |  |  | -0.09 | 0.304 |  | -0.04 | 0.671 |  | -0.01 | 0.898 |  | -0.10 | 0.259 |
| *S. sinesis* | 0.017 | 0.654 | 0.323 |  |  | 0.17 | 0.048 |  | 0.06 | 0.525 |  | 0.05 | 0.536 |  | -0.15 | 0.077 |

^A^ Kruskal-Walli ANOVA
^B^ Spearman rank-order correlation analyses

**Table S3**. A summary of point-value assignment for dietary vegetable (VEG) variety- and frequency-related survey responses used to calculate values for VEG^2^ dietary quality index (DQI). Equations used to calculate VEG^2^ scores for downstream analyses are additionally provided below.

| **VEG Variety**-related survey questions | |  |  | **Yes** | **No** |
| --- | --- | --- | --- | --- | --- |
| 73A. | Did you eat cooked vegetables? |  |  | 1 | 0 |
| 74A. | Did you eat potatoes? |  |  | 1 | 0 |
| 75A. | Did you eat green salads? |  |  | 1 | 0 |
| 76A. | Did you eat organic food? |  |  | 1 | 0 |
|  | **Maximum VEG Variety subtotal** | | | **4** | **-** |
|  |  |  |  |  |  |
| **VEG Frequency**-related survey questions | | **Always** | **Often** | **Sometimes** | **Never** |
| 68. | How often do you have a vegetarian dinner? | 3 | 2 | 1 | 0 |
| 76B. | If (76A = 1), how often do you eat organic food? | 3 | 2 | 1 | 0 |
| 79. | How often do you eat beans, peas, or lentils? | 3 | 2 | 1 | 0 |
| 80. | How often do you eat 2+ vegetables at dinner? | 3 | 2 | 1 | 0 |
| 81. | How often do you eat a vegetable at lunch? | 3 | 2 | 1 | 0 |
|  | **Maximum VEG Frequency subtotal** | **15** | **-** | **-** | **-** |

VEG Variety score = VEG Variety subtotal(sum of subtotals for variety-related questions)

VEG Frequency score = VEG Frequency subtotal(sum of subtotals for frequency-related questions)

VEG^2^_score_=(VEG Variety score) + (VEG Frequency score)(sum of subtotals for variety-related questions)+(sum of subtotals for frequency-related questions)

| **Table S4.** VEG**^2^** quintile distribution data comparison with that of NH participants from MEC using established dietary quality indices (DQI). Data organized by sex in concordance with data from existing literature (Jacobs et al., 2015). | | | | | | | | |
| --- | --- | --- | --- | --- | --- | --- | --- | --- |
|  | [**MEC DQI quintile distribution**](https://www.proquest.com/openview/f9dc9e5c3d0f9333d36454dcd1a9ba7e/1?pq-origsite=gscholar&cbl=48469) **(N)** | | | | |  | **NHPI cohort**  **VEG^2^ (N)** | |
|  | **DASH** | |  | **HEI-2010** | |  |  |  |
|  | **Women** | **Men** |  | **Women** | **Men** |  | **Women** | **Men** |
| **Q1** | 314 | 279 |  | 309 | 226 |  | 14 | 14 |
| **Q2** | 217 | 217 |  | 252 | 221 |  | 17 | 12 |
| **Q3** | 294 | 204 |  | 217 | 195 |  | 15 | 12 |
| **Q4** | 136 | 112 |  | 197 | 163 |  | 16 | 12 |
| **Q5** | 172 | 121 |  | 158 | 128 |  | 15 | 13 |

**Table S5.** Pearson’s chi-square test for independence for cohort quintile distribution of MEC NHPI men and women using existing DQI and that of our NHPI-enriched cohort using the VEG^2^ metric (Jacobs et al., 2015).

|  | **DASH** | | | | |  | **HEI-2010** | | | | |
| --- | --- | --- | --- | --- | --- | --- | --- | --- | --- | --- | --- |
|  | **Women** | |  | **Men** | |  | **Women** | |  | **Men** | |
|  | **𝜒^2^** | **P** |  | **𝜒^2^** | **P** |  | **𝜒^2^** | **P** |  | **𝜒^2^** | **P** |
| **VEG^2^** | 9.2 | **0.056** |  | 7.7 | **0.103** |  | 4.3 | **0.373** |  | 2.7 | **0.606** |

**Table S6.** VEG^2^ correlation with gut microbial phyla, genera and α-diversity.

|  | **VEG^2^** | |
| --- | --- | --- |
|  | **R** | **P** |
| **Age** | -0.17 | 0.051 |
| **A1c** | 0.033 | 0.705 |
| **BMI** | -0.25 | 0.004 |
| **Phylum relative abundance** | | |
| *Actinobacteria* | -0.13 | 0.119 |
| *Bacteroidetes* | -0.06 | 0.464 |
| *Candidatus** | -0.10 | 0.267 |
| *Cyanobacteria* | 0.09 | 0.313 |
| *Deferribacteres* | 0.16 | **0.065** |
| *Elusimicrobia* | -0.01 | 0.935 |
| *Firmicutes* | 0.16 | **0.059** |
| *Fusobacteria* | -0.13 | 0.128 |
| *Lentisphaerae* | -0.12 | 0.155 |
| *Nitrospinae* | -0.04 | 0.639 |
| *Proteobacteria* | -0.08 | 0.336 |
| *Spirochaetes* | 0.05 | 0.589 |
| *Synergistetes* | -0.11 | 0.195 |
| *Tenericutes* | 0.03 | 0.705 |
| *Thermotogae* | 0.08 | 0.378 |
| *Unclassified* | 0.01 | 0.892 |
| *Verrucomicrobia* | -0.20 | **0.018** |
| **Genus relative abundance** | | |
| *Bifidobacterium* | -0.044 | 0.608 |
| *Catenibacterium* | -0.168 | **0.050** |
| *Faecalibacterium* | -0.010 | 0.912 |
| *Lactococcus* | 0.081 | 0.346 |
| *Mucispirillum* | 0.159 | **0.064** |
| *Prevotella* | -0.109 | 0.205 |
| *Ruminococcus* | -0.172 | **0.044** |
| *Serratia* | -0.075 | 0.394 |
| *Shigella* | 0.057 | 0.508 |
| *Streptococcus* | -0.068 | 0.433 |
| **α-Diversity**^C^ |  |  |
| Chao1 | -0.076 | 0.408 |
| Simpson | -0.11 | 0.229 |
| Shannon | -0.13 | 0.156 |
| ^A^ Kruskal-Wallis ANOVA  ^B^ Spearman rank-order correlation coefficient  ^C^ Taxonomic diversity at the genus-level  *Candidatus Thermoplasmatota | | |
